# Supplementary material for: Agent-based modeling of nuclear chromosome ensemble identifies determinants of homolog pairing during meiosis
Source: bioRxiv. 2024 Jan 13:2023.08.09.552574. Preprint. [Version 2] doi: 10.1101/2023.08.09.552574 (PMC10802385; doi:10.1101/2023.08.09.552574)
Supplement: 1 [file NIHPP2023.08.09.552574V2-supplement-1.pdf]

bioRxiv preprint doi: https://doi.org/10.1101/2023.08.09.552574; this version posted January 13, 2024. The copyright holder for this preprint (which was not certified by peer review) is the author/funder, who has granted bioRxiv a license to display the preprint in perpetuity. It is made available under aCC-BY 4.0 International license.

Supporting information

Effect of velocity on pairing kinetics

Similar to Fig. 3, we consider the effect of movement velocity on pairing kinetics. In particular, we observe that pairing kinetics are accelerated as velocity increases. Fig. S1 FigA is a reproduction of Fig. 6A) showing how movement velocity changes the homolog pairing efficiency. Fig. S1 FigB, rescales each curve in (A) by the maximum pairing efficiency. The model predicts that the benefits associated with increased velocity appear to saturate past 300 nm/s. To investigate this trend's direct effect on pairing kinetics, we compute the distances between homologs as a function of chromosome movement speed. The results are summarized in Fig. S2 FigA-D.

bioRxiv preprint doi: https://doi.org/10.1101/2023.08.09.552574; this version posted January 13, 2024. The copyright holder for this preprint (which was not certified by peer review) is the author/funder, who has granted bioRxiv a license to display the preprint in perpetuity. It is made available under aCC-BY 4.0 International license.

Accordingly, smaller compared to larger chromosomes require lower movement velocities for timely pairing completion.

S1 Fig. Effects of chromosome velocity on pairing kinetics. (A) Reproduction of Fig. 6a, each dot indicates the average pairing levels of all 32 chromosomes of true sizes at the indicated time points. Black (300 nm/s) indicates pairing levels at the velocity of chromosome movements in the wild type model in Figs. 3-5 Chromosomes fail to pair at velocities around 150 nm/s, likely due to the effect of thermal noise. Increases in 30 nm/s increments increases pairing efficiencies at $t = 9h \sim 3$-fold, with more modest gains above 240 nm/s where essentially all 16 homologs pair efficiently. (B) Results from Fig. S1A were normalized by maximum pairing levels, as described for Fig. 3B. With increased movement velocities, 50% pairing levels are achieved at progressively earlier time points.

S2 Fig. Effects of movement velocity on homologous pairing kinetics. All results for 16 homolog pairs using true chromosome lengths. Chromosomes are arranged according to their initial distance to facilitate comparison with the experimental data set. Chromosome movement velocity is (A) 180 nm/s, (B) 210 nm/s, (C) 240 nm/s, and (D) 270 nm/s. Note the sharp transition between 210 nm/s and 240 nm/s, as also shown in Fig. 6A. The pairing distance is highlighted in gray at 400 nm. (200 realizations, error bars indicate SD).

Non-homologous chromosome distances in wild type compared to *spo11* hypomorph

The complementary plot to Fig. 7 shows the distribution of non-homologous pairing distances as a function of time. Fig. S3 Fig provides a direct comparison between non-homologous chromosome distances in wild type (A) and *spo11* hypomorph (B) where true chromosome lengths are used. Numerically, we tracked the distance between a given homolog and selected the non-homologous chromosome in the same nucleus with the closest initial distance to its homologous pair. The simulation then tracks the dynamics of both throughout time and we plot the distance between a given chromosome and the previously identified non-homologous chromosome. The results indicate that there is no bias in time for a chromosome with any non-homologous chromosome in the nucleus. Also, the results indicate that the mutation does not have a strong effect on non-homologous interactions as it only modifies their strength, but not their qualitative behavior of creating excluded regions. One difference as noted in Fig. 7 is that while both the wild type and mutant produce excluded regions, the strength of the repulsion in the wild type makes these regions more severe and drives homologous pairing to occur much faster. This validates the model in that the only different interaction occurs with a given chromosome's homologous mate (compare homologous results in Fig. 7 with non-homologous results in Fig. S3 Fig).

S3 Fig. Non-homologous chromosome distances for wild type and *spo11* hypomorph, with experimental observations in the insets. (A) Reproduction of Fig. 4B for non-homologous distances in the wild type simulations. (B) Non-homologous pairs of *spo11* mutants where one of the two homolog partners is matched with a non-homologous chromosome that exhibits an optimally matched initial distance with its cognate homolog partner at $t = 3h$. Inset in (B) shows experimentally determined distances between non-homologous GFP tagged chromosomes II and III. For details on the experimental conditions see [12]. The

bioRxiv preprint doi: https://doi.org/10.1101/2023.08.09.552574; this version posted January 13, 2024. The copyright holder for this preprint (which was not certified by peer review) is the author/funder, who has granted bioRxiv a license to display the preprint in perpetuity. It is made available under aCC-BY 4.0 International license.

pairing distance is highlighted in gray at 400 nm. *Note the experimental data do not include information for $t = 9h$. (200 realizations, error bars indicate SD).

Effect of nucleus size on chromosome kinetics

S4 Fig. Effects of nucleus size on pairing kinetics and efficiency. Average distances over time between homolog pairs to the sizes of actual yeast chromosomes. Here we compare the dynamics as the nucleus size is varied. Observe that pairing occurs much less frequently as the nucleus radius increases. This indicates that confinement and the repulsive non-homologous interactions play a critical role in the kinetics. In a high-density environment like the small nucleus the repulsive interactions have an even larger effect because each chromosome is interacting with many non-homologous neighbors in close proximity. This drives the pairing of homologs by quickly filling the nucleus space with excluded regions due to repulsion. Results over 200 realizations.

Effect of order of magnitude increase/decrease of interaction forces

Here we consider scenarios where either the attractive force strength C_a dominates 100-fold over the repulsive force strength C_r (Fig. S5 FigB) or inversely, the repulsive force strength dominates over the attractive force strength (Fig. S5 FigC). Moreover, with the attractive force dominating, pairing of mid-sized and longer chromosomes occurs essentially instantaneously, whereas the process is drawn out over a longer time scale when repulsive forces dominate.

S5 Fig. Contributions of attractive and repulsive forces on pairing efficiencies and kinetics. (A) Pairing wild-type model using true chromosome lengths and a standard translational movement velocity of 300 nm/s as primarily studied herein. In (B) the repulsive strength is increased by an order of magnitude $C_r = 0.05$ and the attractive strength is decreased by an order of magnitude $C_a = 0.0005$. In (C) the reverse is true $C_r = 0.0005$ and $C_a = 0.05$ (200 realizations, error bars indicate SD). The pairing distance is highlighted by a gray rectangle.

Supplemental movie files

Movie files are available at a dedicated online Zenodo Repository:
`https://zenodo.org/records/10246589`

S1 Video. WT Chromosome Trajectories during Prophase I. The movie shows one realization of the agent-based model. The simulation movie covers the homology search process from $t = 3h$ to $t = 9h$. Matching colors correspond to homologous pairs. True chromosome lengths are incorporated and scale the relevant interaction radii. The radius represents the attractive and non-homologous repulsive region.

S2 Video. WT Chromosome Trajectories during Prophase I with active dumbbell model. The movie shows one realization of the agent-based model active dumbbell model which is closer to modeling a chromosome as a polymer. The simulation movie covers the homology search process from $t = 3h$ to $t = 9h$. Matching colors correspond to homologous pairs. True chromosome lengths are incorporated and scale the relevant interaction radii, but are allowed to change in time as the two beads

bioRxiv preprint doi: https://doi.org/10.1101/2023.08.09.552574; this version posted January 13, 2024. The copyright holder for this preprint (which was not certified by peer review) is the author/funder, who has granted bioRxiv a license to display the preprint in perpetuity. It is made available under aCC-BY 4.0 International license.

expand and contract. The radius represents the attractive and non-homologous repulsive region.

S3 Video. spo11 hypomorph (30% **WT DSB levels) Chromosome Trajectories during Prophase I (parameters from Fig. 7B).** The movie depicts one realization of the agent-based model for the *spo11* hypomorphic mutant. The simulation movie covers the homology search process from $t = 3h$ to $t = 9h$ where mutant *spo11* is associated with a weaker attractive and repulsive force (e.g., reduction to 77% of WT values). True chromosome lengths are incorporated and scale the relevant interaction radii. Matching colors correspond to homologous pairs. The radii represent the homologous attractive and the non-homologous repulsive region. Note that the reduction in interaction strength delays homologous pairing consistent with experimental observations in [12].

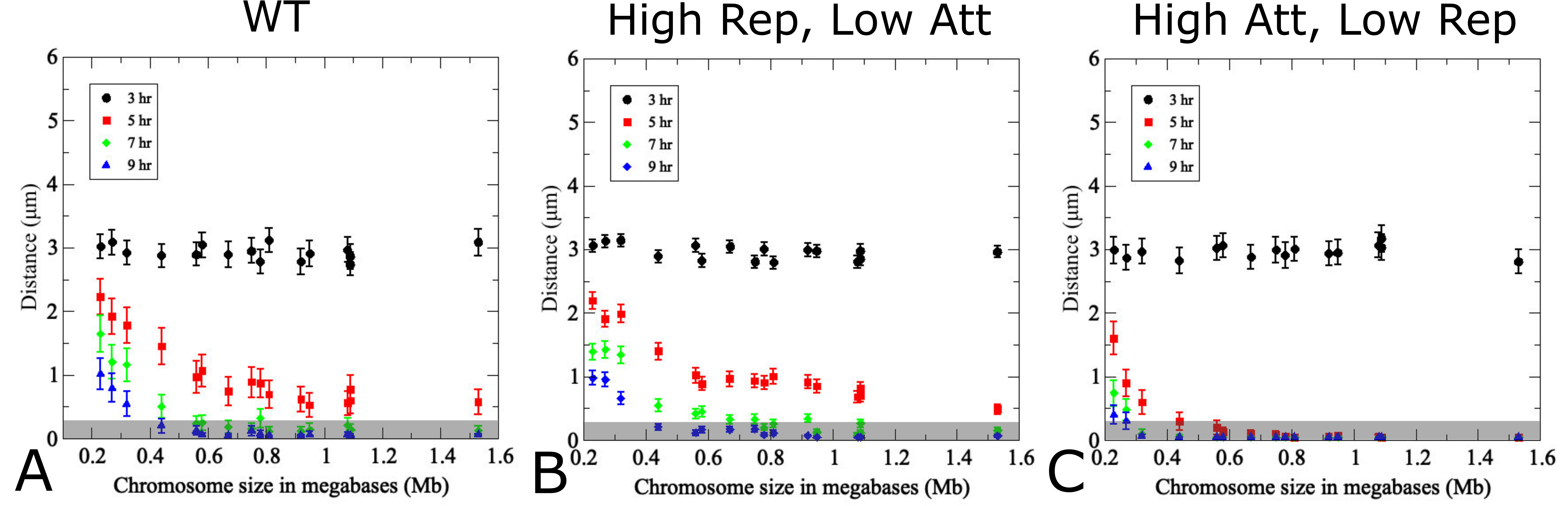

WT
High Rep, Low Att
High Att, Low Rep
3 hr
5 hr
7 hr
9 hr
Distance (μm)
Chromosome size in megabases (Mb)
0
1
2
3
4
5
6
0.2
0.4
0.6
0.8
1
1.2
1.4
1.6
A
B
C

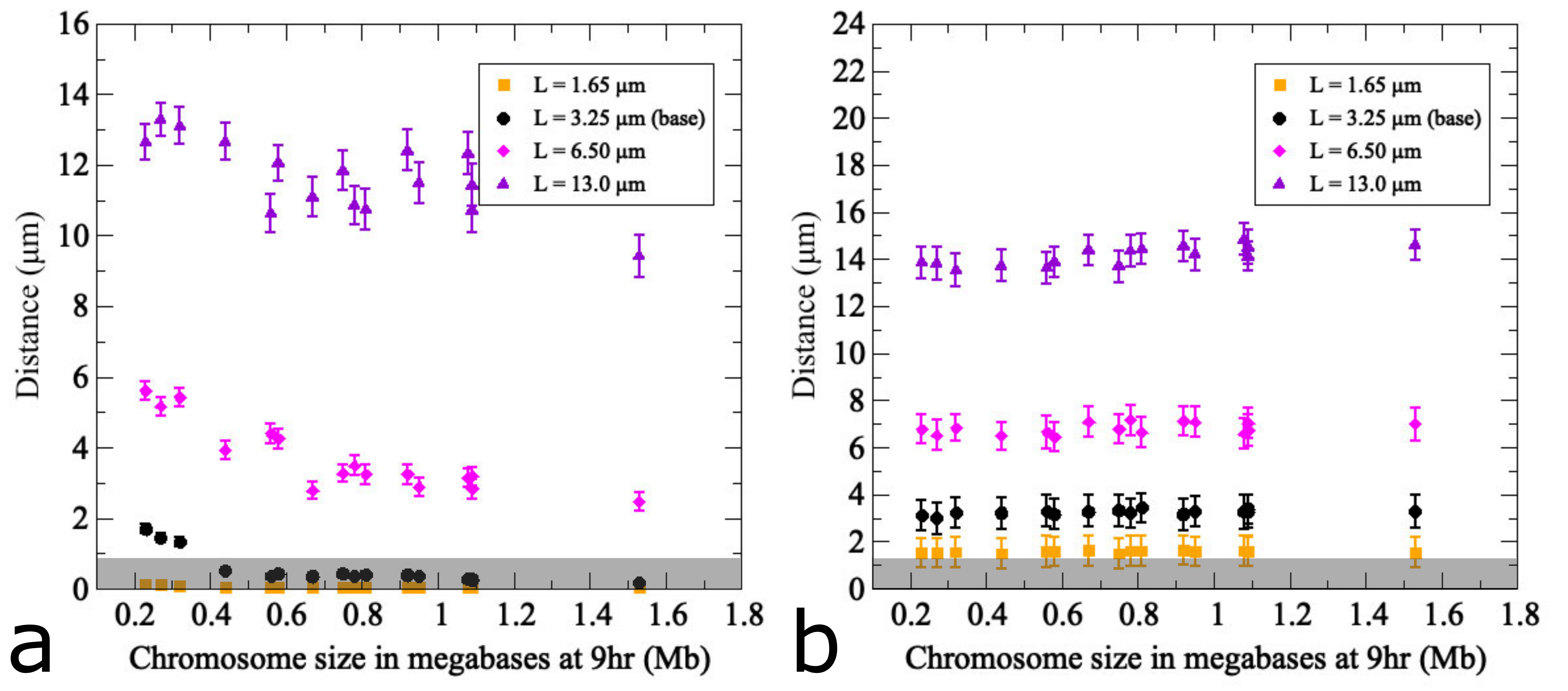

Distance (μm)
Chromosome size in megabases at 9hr (Mb)
L = 1.65 μm
L = 3.25 μm (base)
L = 6.50 μm
L = 13.0 μm
a
b

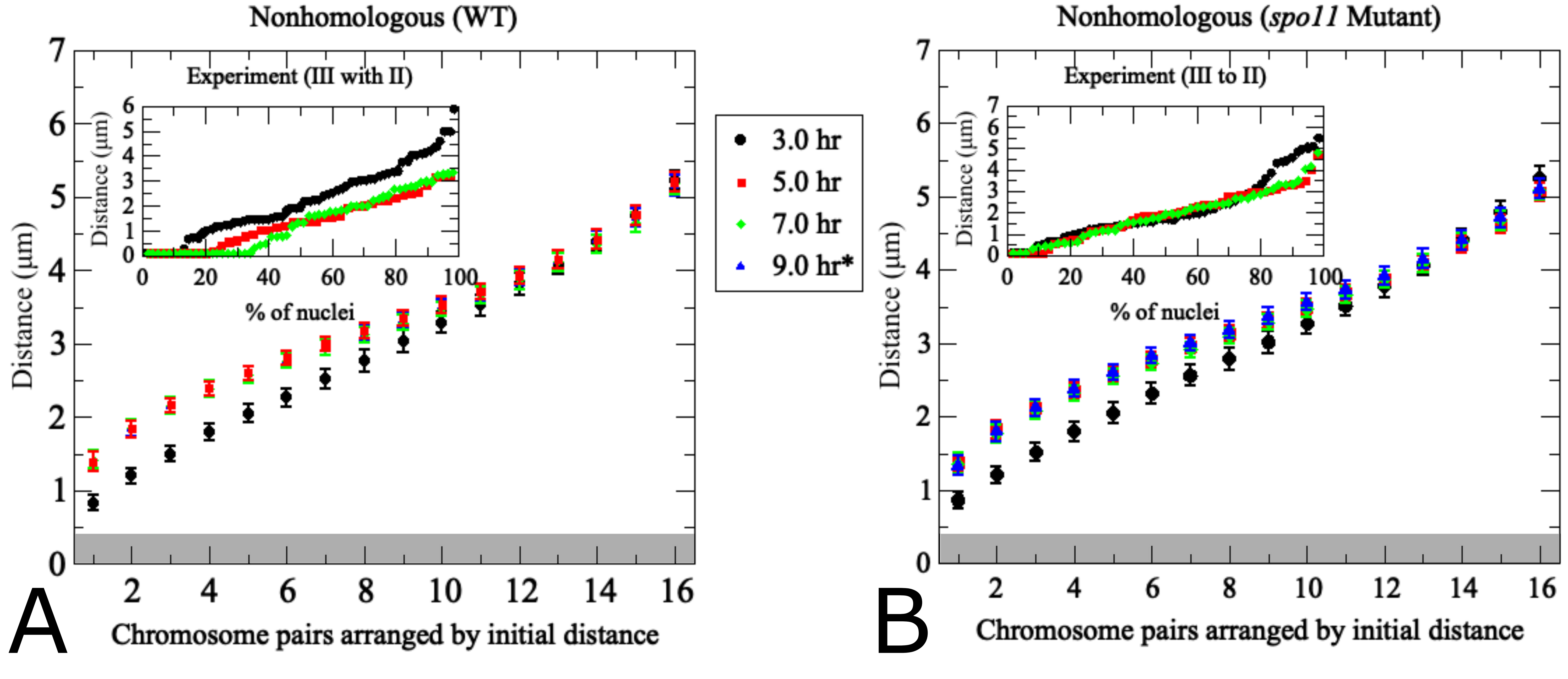

Nonhomologous (WT)
Experiment (III with II)
Distance (μm)
% of nuclei
Chromosome pairs arranged by initial distance
3.0 hr
5.0 hr
7.0 hr
9.0 hr*
Nonhomologous (spo11 Mutant)
Experiment (III to II)
A
B

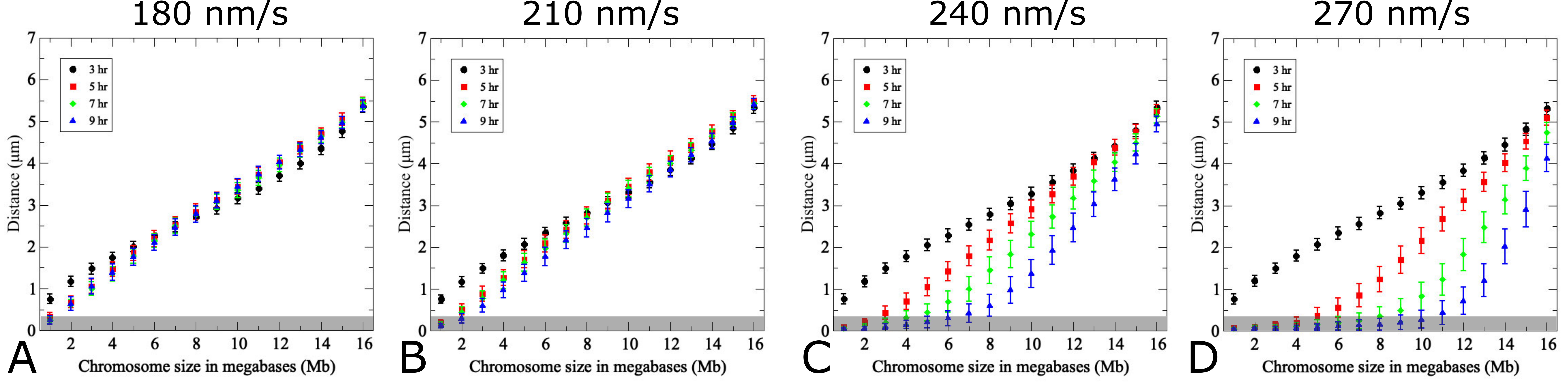

180 nm/s
210 nm/s
240 nm/s
270 nm/s
3 hr
5 hr
7 hr
9 hr
Distance (μm)
Chromosome size in megabases (Mb)
A
B
C
D

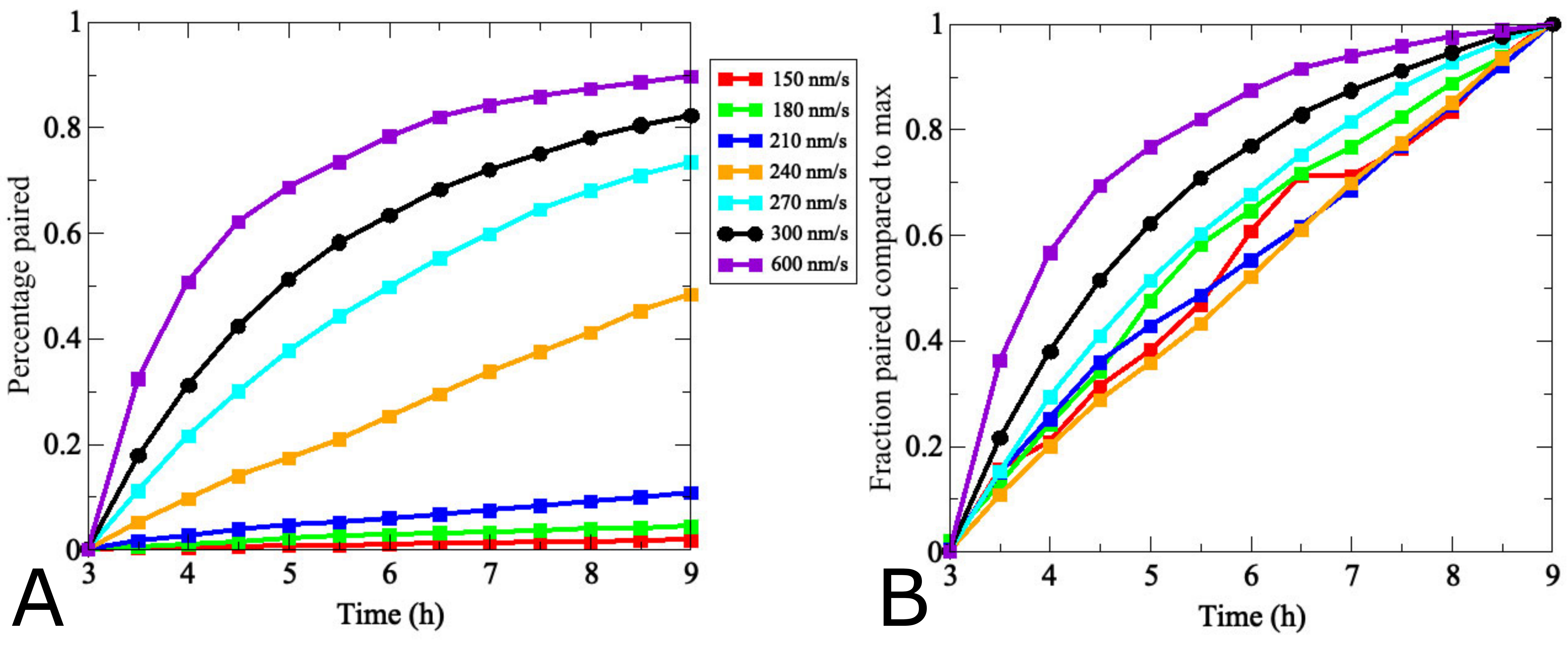
A
Percentage paired
Time (h)
150 nm/s
180 nm/s
210 nm/s
240 nm/s
270 nm/s
300 nm/s
600 nm/s
B
Fraction paired compared to max
Time (h)
